# Oscillometry to assess ventilation heterogeneity during hospital admission for acute cardiorespiratory illness

**DOI:** 10.1183/23120541.00351-2024

**Published:** 2025-06-23

**Authors:** Masarrah Aljaroof, Wadah Ibrahim, Hnin Aung, Rebecca L. Cordell, Michael J. Wilde, Matthew Richardson, Dahlia Salman, Amisha Singapuri, Robert C. Free, Erol Gaillard, Paul Thomas, Paul S. Monks, Christopher E. Brightling, Salman Siddiqui, Neil J. Greening

**Affiliations:** 1 Department of Respiratory Sciences, University of Leicester, Leicester, UK; 2 Institute for Lung Health, NIHR Leicester Biomedical Research Centre (Respiratory theme), Glenfield Hospital, Leicester, UK; 3 Centre for Environmental Health and Sustainability, University of Leicester, Leicester, UK; 4 School of Geography, Earth and Environmental Sciences, University of Plymouth, Plymouth, UK; 5 Bioxhale, Leicester, UK; 6 School of Computing and Mathematical Sciences, University of Leicester, Leicester, UK; 7 School of Chemistry, University of Leicester, Leicester, UK; 8 National Heart and Lung Institute, Imperial College, London, UK; 9 These authors contributed equally

## Abstract

**Rationale:**

Hospitalisation due to exacerbations of cardiorespiratory disease results in reduced lung function and increased airways obstruction. However, traditional measures of lung function require maximal effort which is difficult when patients are unwell (*e.g.* FEV_1_) and may focus on larger airways away from the major part of airways disease (*e.g.* peak flow). We aimed to measure whole airways function using oscillometry in patients during hospitalisation with cardiorespiratory illness compared with healthy controls.

**Method:**

Participants (n=310) underwent assessment; 263 were admitted to hospital with acute cardiorespiratory illness (asthma (n=80), COPD (n=75), heart failure (n=46) and pneumonia (n=62)) and 47 healthy controls were included. Participants underwent handheld oscillometry measurements within the first 24 h of admission.

**Results:**

Oscillometry measurement was feasible in all patients (n=310). There was a significant difference in both absolute and percentage predicted measures of lung mechanics (p<0.05 for all measures), with significantly worse lung mechanics in patients with COPD. Measures of resistance and reactance were worse in those that were more breathless (p<0.0001), had more wheeze (p<0.001) and had low oxygen saturation (p<0.001). No difference was seen based on modified early warning system score or blood biomarkers (eosinophil count, C-reactive protein and brain natriuretic peptide). There were significant improvements in oscillometry measures in those that attended following recovery from acute illness.

**Conclusion:**

Handheld oscillometry can be feasibly deployed in the acute care setting to obtain information on respiratory mechanics. It demonstrates significant differences in ventilation heterogeneity between patients in the acute care setting and healthy volunteers.

## Shareable abstract

**The handheld forced oscillometry technique can be feasibly deployed in the acute care setting to obtain direct information on respiratory mechanics, and is significantly abnormal in acute exacerbations of asthma, COPD, pneumonia and heart failure** https://bit.ly/423i2rH

## Introduction

Hospitalisation due to a cardiorespiratory condition represents a large proportion of unplanned emergency medical admissions. These admissions are commonly due to exacerbations or worsening of chronic conditions, such as asthma, COPD or decompensated heart failure, but may also be due to new acute illness, such as pneumonia [1, 2].

While the systemic severity of the cardiorespiratory illness can be measured through early warning scores and blood biomarkers, no tests are routinely used to directly measure airways function. Symptoms (*i.e.* dyspnoea), oxygen saturation levels (*S*pO_2_) and respiratory rates are collected and provide surrogate markers of function. Traditional measures of airways function, used in the stable state, such as forced expiratory volume in 1 s (FEV_1_), are difficult to implement in the acute setting and are less accurate, as a maximal test is difficult when patients are breathless and fatigued [3]. While peak flow is used in acute asthma, this is a poor measure of lung function, as this provides a measure of more central airways, whereas it is the smaller airways that are more likely to be predominantly affected [4].

Oscillometry is a technique that measures the mechanics of the respiratory system during tidal breathing. It is able to detect both small airways disease and ventilation heterogeneity [5–7]. The technique therefore lends itself to potential use in the acute care setting, where passive ventilation and measures of whole lung mechanics may be of benefit [8]. Despite being described more than 60 years ago by DuBois *et al.* [9], studies of oscillometry during acute severe exacerbations of airways disease, or in other acute cardiorespiratory conditions, are limited, in part as, until recently, oscillometry required highly specialised equipment fixed in a dedicated space. However, more recent oscillometry devices are now portable, allowing bedside measures to be taken [8].

In this study, we describe the feasibility and measures of handheld oscillometry within 24 h of patients being admitted to hospital with acute cardiorespiratory illness due to exacerbations of chronic disease (asthma, COPD, heart failure) or new respiratory illness (pneumonia). We compared these measures across the disease groups to a cohort of healthy volunteers and with symptoms, blood biomarkers and clinical parameters. Finally, we compared measures of handheld oscillometry in a subset of participants who returned once recovered.

## Methods

### Study design and participants

This was a prospective cohort study of patients admitted to hospital with self-reported acute breathlessness and a primary clinical diagnosis of one of: exacerbation of asthma, exacerbation of COPD, decompensated heart failure or pneumonia.

Participants were prospectively recruited between May 2017 and December 2018, as part of the Exhaled Breath Metabolomic Biomarkers in the Acutely Breathless Patient (EMBER) study [10, 11], for which oscillometry was collected as a pre-determined secondary outcome.

Informed written consents were obtained and measures, including oscillometry, performed within 24 h of hospital admission. Age-matched healthy controls were also recruited, with no history of prior cardiorespiratory illness. Acute participants were invited back for a second visit, once recovered (at least 6 weeks), for repeat measures. Full details of the protocol and inclusion/exclusion criteria have previously been published [11]. The study was approved by the National Research Ethics Service Committee (16/LO/1747).

### Oscillometry

Participants performed handheld oscillometry measurements using TremoFlo airway oscillometry (Tremoflo C-100, Thorasys, Canada) in line with the American Thoracic Society/European Respiratory Society (ATS/ERS) consensus [12, 13]. The Tremoflo is a United States Food and Drug Administration (FDA) approved, oscillometry device, which has previously shown good concordance with impulse oscillometry [14]. The oscillometry device was calibrated daily using standardised volume and resistance. Oscillometry was performed with participants sitting up with the head in a neutral or slightly extended position. Participants were instructed to form a tight seal around the mouthpiece and to firmly use both hands to suppress their cheeks to minimise airway shunting. Nose clips were worn during the procedure. Pressure oscillations were initiated and subjects were instructed to breathe quietly at the functional residual capacity level. A total of three technically acceptable measurements were performed.

### Oscillometry parameters and normative values

Several oscillometry measurements of lung mechanics were collected in this study. For the resistive component of the respiratory impedance, we measured the resistance at 5 Hz (R5), at 19 Hz (R19), and the resistance at 19 Hz minus the resistance at 5 Hz (R5–R19). These measurements, respectively, indicate the resistance of the whole respiratory system, the proximal airways and primarily the small airway plus the ventilation inhomogeneities of the system. Airways resistance from impulse oscillometry has been shown to be related to heterogeneous lung ventilation using quantitative computed tomography [15]. To reflect on the reactance component of the respiratory impedance, we collected the data for the reactance at 5 Hz (X5) and the area under the reactance curve (AX), which are both measurements of the elastic properties, lung stiffness and the existing heterogeneous ventilation. Moreover, we reported the resonant frequency (Fres), where inertance and elastance are balanced against each other and the whole impedance is explained by resistance.

Oscillometry measurements of lung mechanics are influenced by the subject's unique characteristics (including age, gender, height, weight and body mass index (BMI)) [16–18]. Several studies have published reference equations for the percentage predicted value to correct for these factors using oscillometry techniques. However, considerable variation exists between cohorts and it is recommended that the reference normal values are generated based on the equation that best matches both the characteristics of the targeted population and measurement techniques [19]. We generated predicted values equations based on the recruited group of healthy control volunteers to best match the population of interest (see supplementary table S3).

### Statistical analysis

Baseline characteristics, oscillometry measures between disease groups, and across clinical and blood biomarker groups at baseline, were compared using one-way analysis of variance (ANOVA) and Kruskal–Wallis for parametric and nonparametric data, respectively. Post-hoc Dunn's test was applied for multiple comparisons. A sub-analysis was performed in nonsmokers (ex-smokers or never-smokers). Chi squared tests were used for categorical data. Spearman Rho was used for correlation coefficients. Linear regression and logistic regression were used to look at measures of oscillometry and length of hospital stay and hospital readmission, respectively. Paired, longitudinal data were analysed using the Wilcoxon signed rank test.

For the oscillometry normative values, linear regression models were used to adjust for the subject's height and BMI as predictors for each oscillometry measure. The effect of age, weight, gender, height and BMI were examined initially as independent variables in the regression models, but, subsequently, age, gender, and weight were excluded as these variables were insignificant in the model (see supplementary table S4).

## Results

### Feasibility of using oscillometry in acute settings

Oscillometry was attempted in 310 out of 386 (80.3%) participants from the EMBER study, with the device not being available for the 76 participants in which oscillometry was not attempted. Oscillometry was successful in 310 out of 310 (100%) participants in obtaining data to ATS/ERS standard. No adverse events were noted during or after the oscillometry manoeuvre.

### Baseline characteristics

In total, 263 out of 310 (84.8%) participants were admitted with self-reported acute breathlessness with an exacerbation of asthma (n=80), acute exacerbation of COPD (n=75), decompensated heart failure (n=46) or pneumonia (n=62). Presenting dyspnoea, measured using the visual analogue score, was similar across all disease groups and was significantly higher than in healthy controls (p<0.001). Participants with asthma were younger and more likely to be female than other groups. Patients with COPD were more likely to be current smokers than other groups, and BMI was highest in the asthma and heart failure groups. Full clinical observations and laboratory blood results are presented in table 1.

**TABLE 1:** Baseline characteristics

	Healthy	Asthma	COPD	Pneumonia	Heart failure	p-value
Total (n)	47	80	75	62	46	
**Demographics**						
Age, years	63.1±12.1	41.7±17.5	68.9±8.3	59.0±16.9	72.4±10.42	<0.001
Male sex, n (%)	25 (49%)	33 (41%)	45 (60%)	30 (48%)	32 (70%)	0.021
Height, m	1.69±0.11	1.67±0.11	1.67±0.09	1.69±0.09	1.70±0.11	0.457
Weight, Kg	79.3±16.0	86.7±25.5	74.9±22.4	81.6±21.7	91.4±26.2	0.001
BMI, Kg·m^−2^	27.6±4.2	31.0±9.1	26.7±7.4	28.8±7.79	31.2±7.2	0.001
Current smoker	0 (0%)	21 (26%)	33 (44%)	13 (21%)	6 (13%)	<0.001
**Clinical observations**						
Temperature, °C	36.2±0.4	36.9±0.6	36.8±0.52	37.3±0.8	36.6±0.4	<0.001
Heart rate (beats·min^−1^)	66±14	98±18	92±18	93±16	80±15	<0.001
Respiratory rate (breath ·min^−1^)	13.5±2.9	20.8±3.2	21.9±9.5	21.3±10.3	20.2±3.8	<0.001
Oxygen saturation (*S*pO_2_) (%)	98±1	96±2	94±3	95±4	97±2	<0.001
Systolic blood pressure (mmHg)	134±18	134±19	133±21	128±20	126±21	0.103
**MEWS score**	1±1	2±2	3±3	2±2	2±2	<0.001
**Dyspnoea measures**						
MRC score	1.0 (1.0–1.0)	5.0 (4.0–5.0)	5.0 (4.0–5.0)	5.0 (3.0–5.0)	5.0 (4.0–5.0)	0.039
Breathlessness VAS score (mm)	3.48±(5.33)	69.76±(21.76)	69.53±(18.63)	67.7±(22.5)	68.8±(18.2)	<0.001
Wheeze VAS score (mm)	2.98±(5.27)	65.72±(23.10)	59.28±(28.15)	43.62±(33)	30.02±(29.5)	<0.001
**Laboratory**						
Eosinophil count × 10^9^·L^−1^	0.14 (0.09–0.21)	0.17 (0.06–0.40)	0.14 (0.06–0.30)	0.10 (0.05–0.14)	0.12 (0.07–0.22)	<0.001
Brain natriuretic peptide (ng·l^−1^)	15.0 (18.2–40.7)	20.2 (7.7–39.7)	50.5 (25.1–92.4)	12.5 (25.6–122.5)	520.8 (175.4–1051.5)	<0.001
C-reactive protein (mg·L^−1^)	2.5 (2.5–2.5)	8.0 (2.5–20.0)	12.0 (2.5–25.75)	149.0 (84.0–252.0)	13.0 (2.5–24.0)	<0.001

Data are expressed as mean±sd, median (interquartile range) or proportions (%). BMI, body mass index; MEWS, modified early warning system; MRC, Medical Research Council Dyspnoea scale; VAS, visual analogue scale (100 mm).

### Measures of lung mechanics during acute illness

**FIGURE 1:**
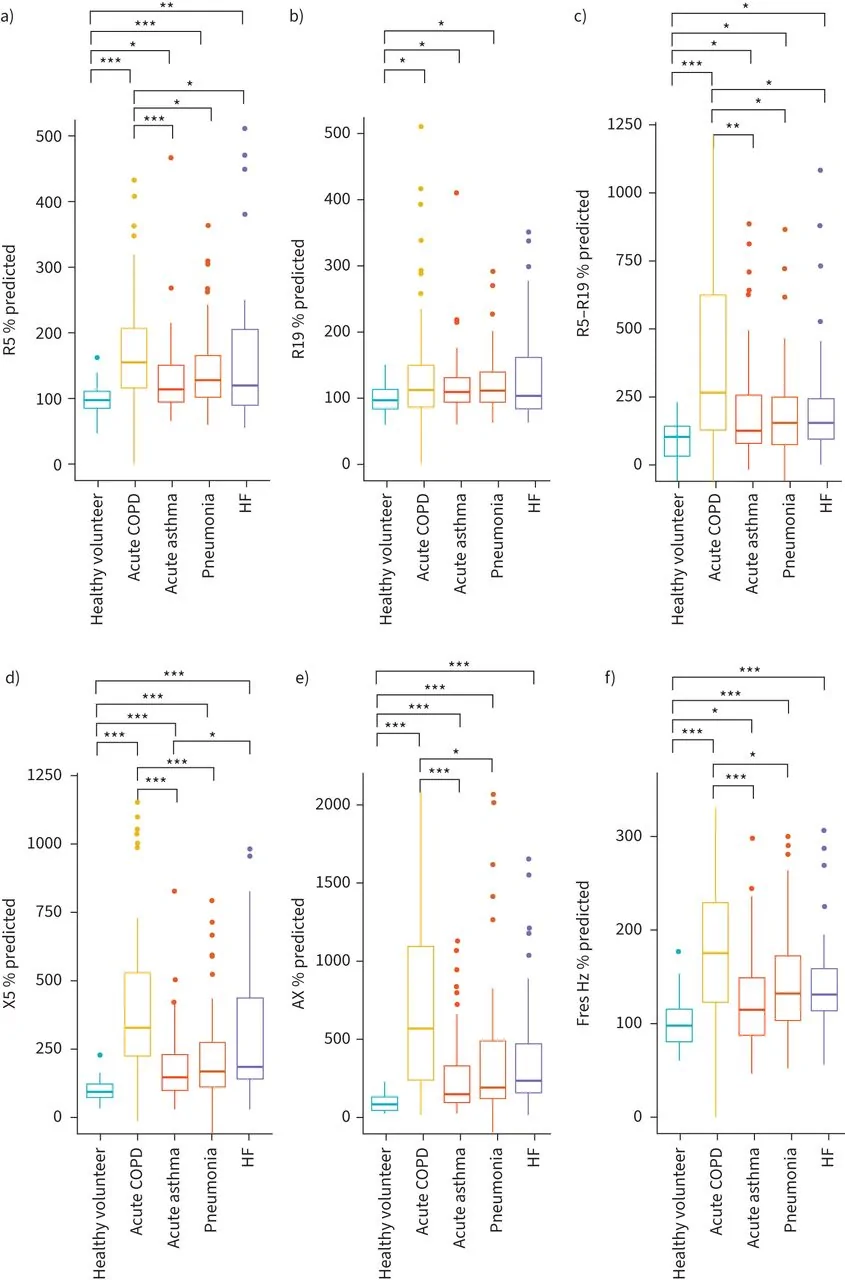
Box and whisker plots of % predicted measures of lung mechanics in healthy volunteers and acutely unwell patients with COPD, asthma, pneumonia and heart failure. (a) R5 % predicted (b) R19 % predicted (c) R5−R19 % predicted (d) X5 % predicted (e) AX % predicted (f) Fres % predicted. *p<0.01, **p<0.001, ***p<0.0001. Lines represent medians; boxes represent IQRs; whiskers represent 5–95% confidence intervals and outliers are represented as circles. AX: area above the reactance curve from 5 Hz to resonant frequency; Fres: resonant frequency; HF: heart failure; IQR: interquartile range; R5: respiratory resistance at 5 Hz; R19: respiratory resistance at 19 Hz; R5−R19: difference in resistance between R5 and R19; X5: respiratory reactance at 5 Hz.

All measures of lung mechanics using oscillometry were significant higher in participants hospitalised with breathlessness than healthy volunteers, irrespective of aetiology (table 2). The only measure for which no significant difference was seen between healthy volunteers and patients with heart failure was R19 (figure 1b). The greatest differences, compared with healthy volunteers, were seen in R5−R19, X5 and AX (figure 1c, d and e). Patients with an acute exacerbation of COPD also had significantly higher abnormal lung mechanics (R5, R5−R19, X5, AX and Fres) than other acute conditions (figure 1). In nonsmokers (n=73 of the acute cohort), there were significant differences in all measures of oscillometry between healthy volunteers and each disease group.

**TABLE 2:** Measures of oscillometry in healthy controls and acutely unwell patients with different cardiorespiratory conditions

	Healthy	Asthma	COPD	Pneumonia	Heart failure	p-value
**Total (n)**	47	80	75	62	46	
**R5 (kPa.s.L^−1^)**	3.70 (3.115–4.810)	5.28 (3.84–7.16)	5.61 (4.62–7.85)	5.11 (4.077–6.910)	5.44 (4.84–6.978)	<0.001
**R19 (kPa·s·L^−1^)**	3.10 (2.690–3.785)	3.85 (3.067–5.008)	3.62 (2.89–5.135)	3.82 (3.038–4.612)	3.81 (2.895–5.418)	0.014
**R5–R19 (kPa·s·L^−1^)**	0.61 (0.215–1.135)	1.23 (0.46–2.16)	2.14 (1.49–2.715)	1.35 (0.815–2.237)	1.81 (1.32–2.28)	<0.001
**X5 (kPa·s·L^−1^)**	−1.48 (−2.045–−1.050)	−2.61 (−3.893–−1.448)	−5.08 (−7.285–−3.275)	−2.68 (−4.838–1.750)	−4.04 (−7.15–−2.627)	<0.001
**AX (kPa·L^−1^)**	7.53 (3.895–15.200)	20.45 (8.828–46.828)	55.25 (33.03–85.08)	25.27 (13.18–54.87)	37.35 (27.12–64.07)	<0.001
**Fres (Hz)**	23.00 (12.62–21.91)	40.50 (16.63–28.78)	37.00 (27.18–33.21)	31.50 (21.92–30.47)	23.50 (23.67–29.55)	<0.001

Data are presented as median (interquartile range). AX: area above the reactance curve from 5 Hz to resonant frequency; Fres: resonant frequency; R5:  respiratory resistance at 5 Hz; R19: respiratory resistance at 19 Hz; R5−R19: difference in resistance between R5 and R19; X5: respiratory reactance at 5 Hz.

### Association between lung mechanics, clinical parameters and blood biomarkers

Oscillometry was compared with symptoms (breathlessness and wheeze) and routinely collected clinical parameters used to monitor the respiratory system: oxygen saturation (*S*pO_2_), respiratory rate and modified early warning system (MEWS) score. Measures were either poorly, or not significantly, correlated, with measures of oscillometry (see supplementary table S6). *S*pO_2_ and Fres had the highest correlation coefficient (−0.35, p<0.001).

**FIGURE 2:**
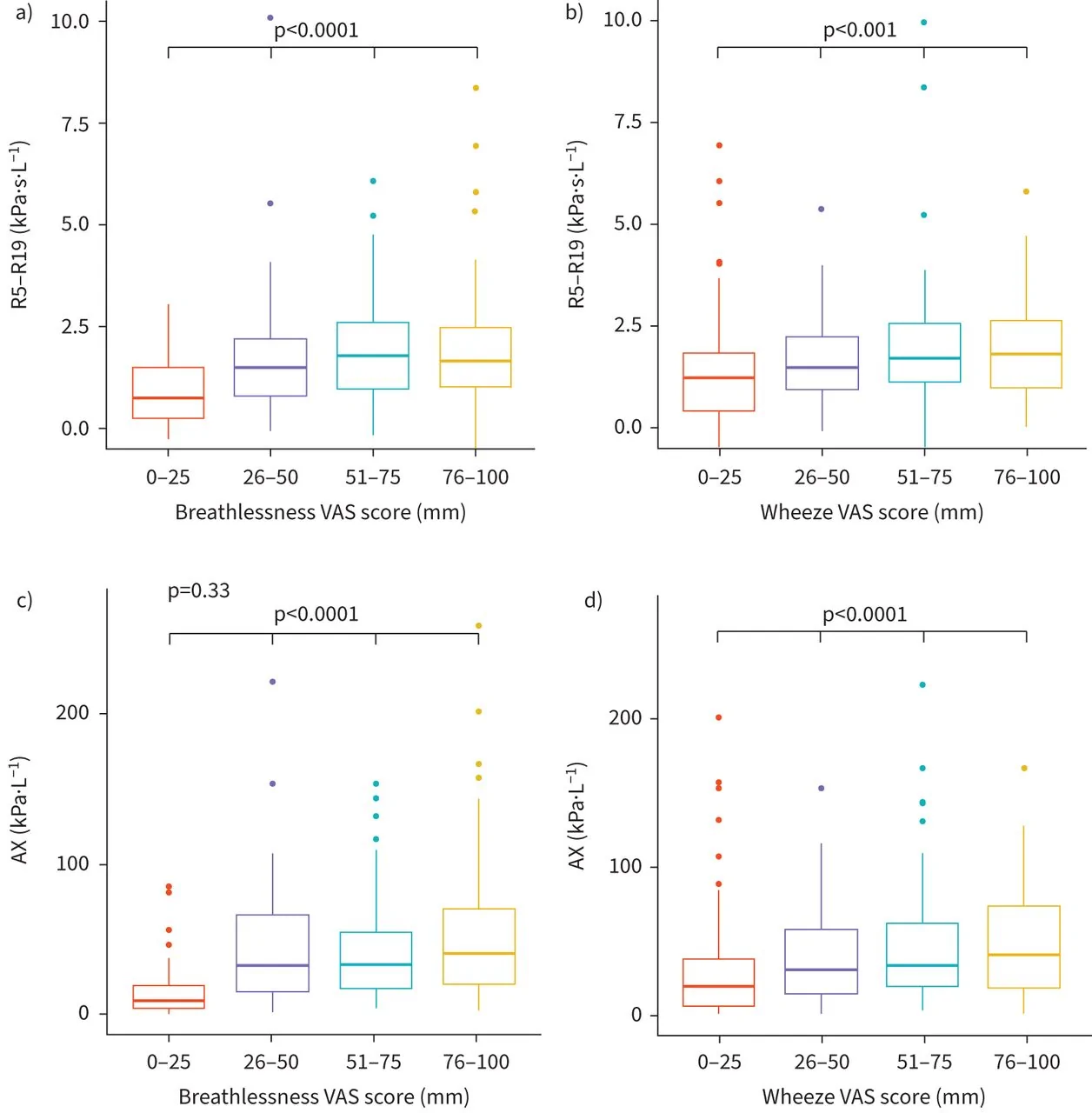
Box and whisker plots of selected lung mechanics representing resistance and reactance comparing breathlessness and wheeze using VAS where higher score represents worse symptoms. (a) R5−R19 across breathlessness, (b) R5−R19 across wheeze, (c) AX across breathlessness, (d) AX across wheeze. AX: area above the reactance curve from 5 Hz to resonant frequency; R5−R19: difference in resistance between R5 and R19; VAS: visual analogue score; X5: respiratory reactance at 5 Hz.

**FIGURE 3:**
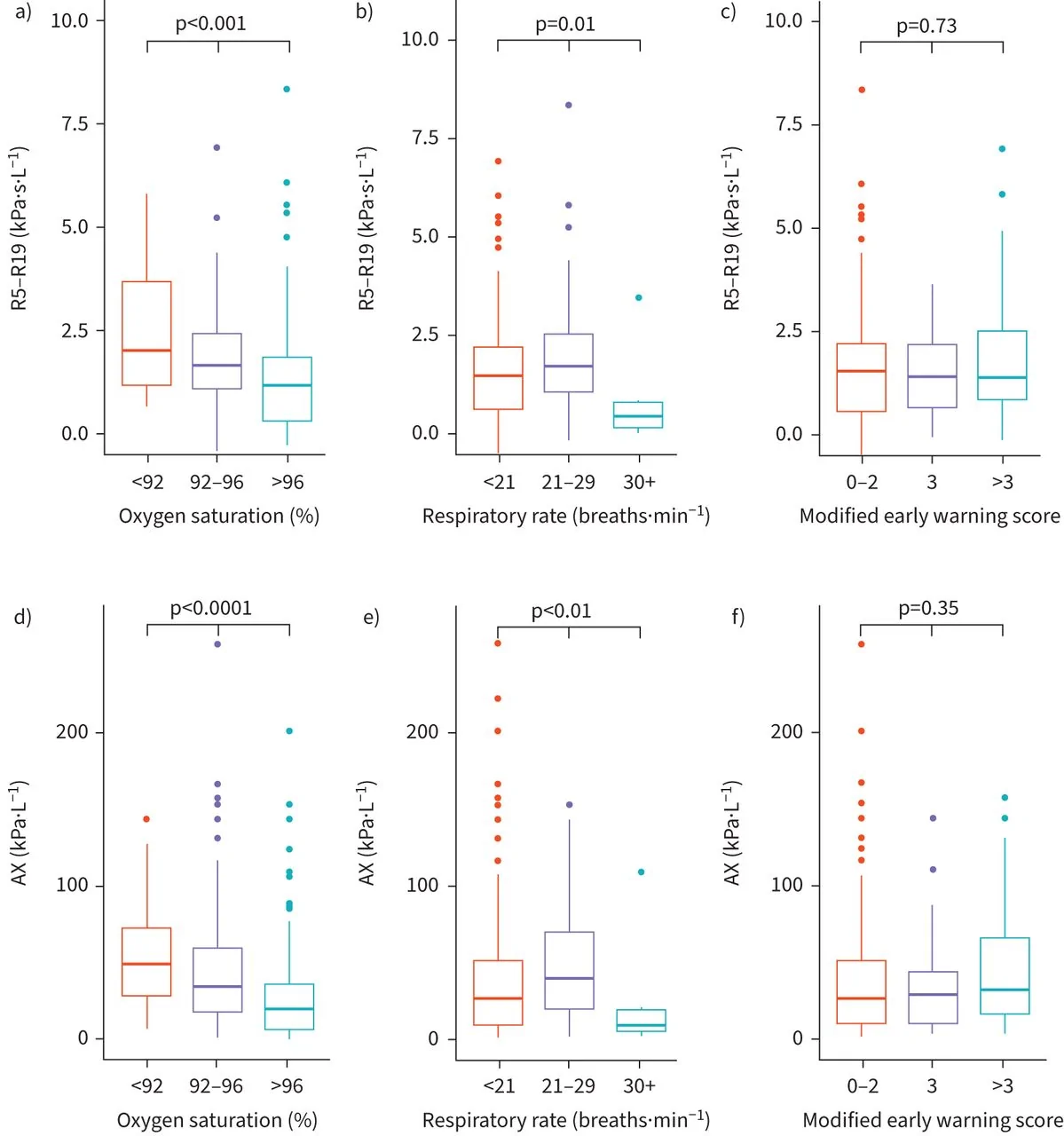
Box and whisker plots of selected lung mechanics representing resistance and reactance *S*pO_2_, respiratory rate and MEWS score. (a) R5−R19 across *S*pO_2,_ (b) R5−R19 across respiratory rate, (c) R5−R19 across MEWS score, (d) AX across *S*pO_2,_ (e) AX across respiratory rate, (f) AX across MEWS score. AX: area above the reactance curve from 5 Hz to resonant frequency; MEWS: modified early warning system; R5−R19: difference in resistance between R5 and R19; *S*pO_2:_ oxygen saturation.

Measures of lung mechanics were significantly different with increasing breathlessness and wheeze (figure 2 and supplementary table S7). Similarly, worse lung mechanics were seen in those who were hypoxaemic (*S*pO_2_ <92%) (figure 3a and d and supplementary table S7). While there were significant differences between lung mechanics and respiratory rate, this was seen in those with a respiratory rate of >30 breaths per minute (figure 3b and e) and no difference was seen based on the MEWS score. No difference was seen in lung mechanics between high and low blood biomarkers of eosinophils count, C-reactive protein (CRP) or pro B-type natriuretic peptide (pro-NT BNP) (supplementary figure S1 and table S7).

### Lung mechanics and healthcare utilisation

Oscillometry at time of hospital admission did not predict length of hospital stay, nor hospital readmission at 30 or 90 days (p>0.05 for all measures of oscillometry and outcomes, data not shown).

### Longitudinal follow-up

**FIGURE 4:**
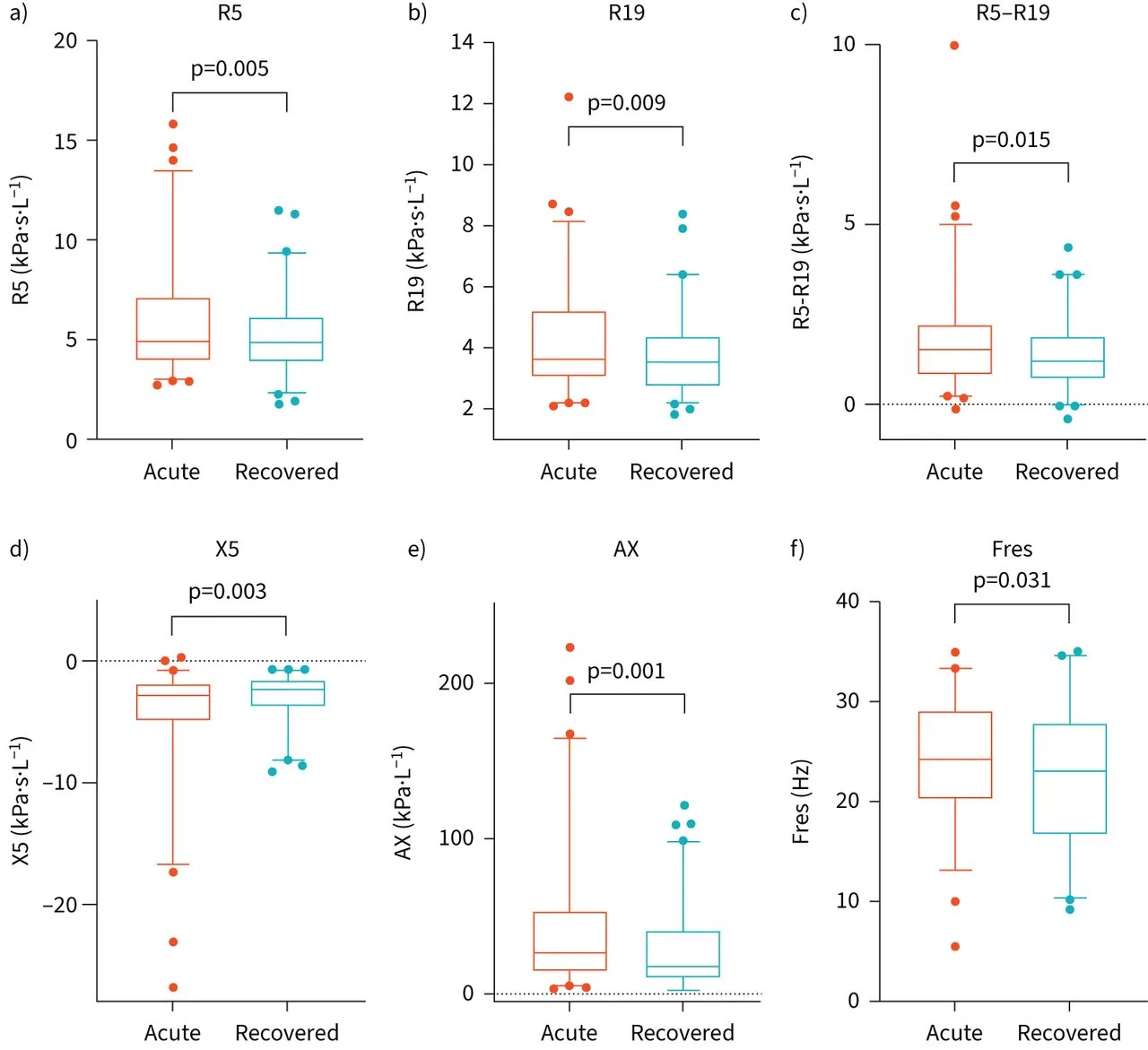
Box and whisker plots of lung mechanics comparing participants in the acutely unwell state and following recovery (n=63). (a) R5, (b) R19, (c) R5–R19, (d) X5, (e) AX, (f) Fres. Lines represent medians; boxes represent IQRs; whiskers represent 5–95% confidence intervals and outliers are represented as circles. AX: area above the reactance curve from 5 Hz to resonant frequency; Fres: resonant frequency; IQR: interquartile range; R5: respiratory resistance at 5 Hz; R19: respiratory resistance at 19 Hz; R5−R19: difference in resistance between R5 and R19; X5: respiratory reactance at 5 Hz.

Once they felt recovered, 63 (24%) of acute participants attended a follow-up visit. These included patients with asthma (n=19), patients with COPD (n=20), patients with heart failure (n=11) and patients with pneumonia (n=13). A comparison of demographics between those that did and did not attend the recovery visit is shown in supplementary table S8. Significant improvement was seen between acute and recovered states in all measures of lung mechanics (figure 4). Significant improvements were also seen in participants who had a chronic disease (*i.e.* asthma, COPD or heart failure) (all p<0.05). Paired changes in R5−R19 and AX for individuals for each disease state are shown in supplementary figure S2.

## Discussion

In this prospective observational study of patients hospitalised with acute breathlessness due to COPD, asthma, pneumonia or heart failure, we demonstrated that lung mechanics measured using oscillometry are abnormal in all parameters compared with healthy volunteers. These measures are most abnormal in those with acute exacerbations of COPD, compared with exacerbations of asthma, pneumonia and heart failure. We also showed that lung mechanics are related to symptoms of breathlessness and wheeze, as well as oxygen saturation. This supports the use of oscillometry in the acute care setting as a direct measure of lung mechanics and as a potential clinical tool. Finally, we demonstrated that measures of oscillometry improve following recovery from their acute illness, including those with chronic disease.

We observed differences in acute breathlessness in measures of both resistance and reactance. This would suggest abnormalities in the acute care setting occur in both the small airways disease and lung elasticity, and are not specific to one aspect of lung mechanics. In addition, changes in both resistance and reactance were similar across different aetiologies of breathlessness, despite different underlying pathophysiologies. This is unsurprising as the cardiorespiratory diseases in this cohort will cause lung infiltrates irrespective of aetiology. This means that oscillometry is unlikely to be able to be used to diagnose the aetiology of dyspnoea; however, it could be used as a tool to monitor the respiratory system independent of cause.

Worse respiratory symptoms and low oxygen saturation were associated with abnormal measures of oscillometry. Conditions with increased airway resistance or reduced lung compliance can cause increased effort by the respiratory muscles to move air in and out of the lungs, resulting in increased energy expenditure and oxygen consumption. If the lungs cannot adequately meet this increased demand, irrespective of the underlying pathophysiology due to compromised respiratory function, this may lead to decreased oxygen saturation levels in the blood (hypoxaemia). V/Q mismatch occurs when ventilation and perfusion in different areas of the lungs are not well matched, leading to impaired gas exchange. Increased airway resistance can limit airflow to certain regions of the lungs, contributing to V/Q mismatch, while reduced lung compliance can make it more difficult to move air in and out of the lungs, thus same respiratory driving pressure will result in lower minute ventilation compared with normal lung.

The presence of more negative values of X5 (indicating lower elastance) and higher values of R5−R19 or AX during acute exacerbations in respiratory conditions can be attributed to a combination of factors such as mucous plugging, airway closure and bronchoconstriction. Exacerbations are often accompanied by elevated levels of lung extracellular water (pulmonary oedema) in patients with heart failure.

Oscillometry had poor correlations with clinical parameters, meaning that oscillometry may add additional information beyond clinical observation *i.e.* an indicate of compromised respiratory function that may not be apparent through clinical examination reflected by airway resistance, lung compliance and ventilation homogeneity. Thus, oscillometry could help to provide insights to healthcare provides into the underlying dysfunction associated with these respiratory mechanisms, such as V/Q mismatch, physiological shunting and increased work of breathing, which may not be readily apparent through clinical observation. This additional information can potentially help in developing more targeted treatment plans and optimising patient care, including site of monitoring (*e.g.* respiratory support unit and high dependency units).

A respiratory rate of greater than 30 was associated with lower measures of oscillometry. An increased respiratory rate is known to be a marker of disease severity, and therefore we expected to see worse measures of oscillometry. The observations of those with a respiratory rate of greater than 30 may be due to inaccuracies in oscillometry measurements at higher rates, where tidal volume is less likely to be achieved. Severe tachypnoea (above 30 breaths per minute in this case), typically characterised by shallow breathing involving decreased depth and force of breaths, reduced turbulence in the airflow at lower frequencies, decreased tidal volume, minute ventilation and lung overdistention leading to a drop in the measured resistive and reactive component of respiratory impedance.

It is important to validate this observation if oscillometry is to be used clinically in the acute care setting, as it may underestimate poor lung mechanics.

This is the first study to study oscillometry across different multiple cardiorespiratory conditions in the acute hospital setting. A small number of studies have looked at individual conditions in patients in hospital, such as COPD [20, 21] and asthma [22], mostly in the paediatric population. Studies have consistently shown that the measure is feasible in the acutely unwell patient, though have not previously explored differences in clinical parameters. One other study has compared patients with COPD and patients with heart failure, and similar to our findings, noted worse lung mechanics in patients with COPD [21].

This study looked at oscillometry at the time of acute admission to hospital; however, it did not look at recovery during the acute phase. The feasibility and speed of oscillometry means that regular monitoring would be feasible and may be able to accurately track clinical recovery and when it would be safe for hospital discharge, much like peak flow is used for asthma guidelines. Future, prospective studies here would be of interest. However, in the participants who attended for a visit following recovery, measures had significantly improved. This suggests that the abnormalities seen were, at least in part, due to acute changes.

In this paper, we have identified changes in both resistance and reactance that distinguish the acute exacerbation group from the healthy group using traditional respiratory impedance measurements (Zrs), which fails to capture certain respiratory occurrences such as dynamic changes in lung compliance, variations in bronchial airway resistance at different lung volumes, and changes in airway resistance in response to variations in airflow rate or magnitude during breathing. Recent advancements in oscillometry have introduced new modalities and measures, including intra-breath measures of respiratory impedance and airway impedance entropy, to overcome these limitations [23, 24].

Limitations of the study include that oscillometry was not the primary outcome of the study, though was a prespecified outcome measure. As discussed above, the measure was only taken at time of admission and not longitudinally. The largest limitation to the use of oscillometry is the inter-person variability of the measure and that accurate normative values in this study population do not exist. However, this is currently underway and would allow more standardised future studies. Finally, only a subset of participants attended the recovery visit, so changes between the acute and stable states were underpowered.

In summary, measures of lung mechanics using oscillometry are feasible across patients admitted to hospital with breathlessness due to cardiorespiratory conditions. These patients are significantly different to healthy controls, with the worst measures seen in patients with COPD. Oscillometry is related to measures of symptoms and respiratory failure and offers a direct measure of lung mechanics.

## Supplementary material

**Please note:** supplementary material is not edited by the Editorial Office, and is uploaded as it has been supplied by the author. Supplementary material 00351-2024.SUPPLEMENT
