# Supplementary material for: Oscillometry to assess ventilation heterogeneity during hospital admission for acute cardiorespiratory illness
Source: ERJ Open Res. 2025 Jun 23;11(3):00351-2024. doi: 10.1183/23120541.00351-2024 (PMC12183746; doi:10.1183/23120541.00351-2024)
Supplement: Supplementary file 1 [file 00351-2024.SUPPLEMENT.pdf]

Online Supplement

Tables S1: *Demographics and baseline Oscillometry data for the healthy subjects included for reference values calculation.*

Total (43)	Min	Max	1st Qu	Median	Mean	3rd Qu
Sex Male, n (%)	22 (% 51.2)					
Age (years)	50	80	60.50	66	65.35	70.50
Height (m)	1.49	1.91	1.62	1.68	1.69	1.77
Wight (Kg)	45.0	111.1	66.6	78.9	78.5	88.00
BMI (Kg/m2)	20.5	34.8	24.7	27.1	27.3	29.8
Measured R5	1.70	7.05	3.11	3.70	3.94	4.64
Measured R19	1.90	5.31	2.67	3.10	3.27	3.77
Measured R5-R19	-0.28	2.92	0.21	0.49	0.67	1.02
Measured X5	-3.85	-0.49	-2.04	-1.50	-1.63	-1.05
Measured Ax	0.84	28.22	3.89	7.53	10.26	15.20
Measured fres Hz	8.37	29.27	12.62	16.11	17.04	20.93

Table S2: *Comparison of healthy subjects included for reference values calculation in current work vs. in published reference equations by In Oostveen E.et al*[1]

	In current study	In *Oostveen E.et al*
Total	43	368
Age (years)	65.35 ± (7.76)	49 ±17
Sex Male, n (%)	22 (% 51.2)	(49%)
Height (m)	1.69 ± (0.11)	1.71 ± (0.9)
Wight (Kg)	78.55 ± (15.78)	n/a
BMI (Kg/m2)	27.29 ± (3.87)	25.5 ± 3.94
Model Specifications	Multiple linear regression<br><br>Height and BMI as predictors, age not sign in all models<br><br>Used FOT, one center.	Mixed model analysis<br><br>Age, height, and weight as predictors.BMI not sign in all models, age sign in some was not excluded for the purpose of consistency<br><br>Used FOT and IOS techniques including 3 commercial and 2 custom- made FOT devices in 5 centres

.

Tables S3: *Univariate analysis using linear regression for each measure. Age and weight were not related to the measured FOT parameter in all models in this particular group of subjects while gender, height, BMI found to be significant and included in subsequent multivariate analysis.*

**Predictor**	**Age**		
**(Rrs) or (Xrs)**	**Beta Coefficient**	**95 % CI**	**P value**
**R5**	0.006	[-0.044, 0.056]	0.806
**R19**	-0.008	[-0.042, 0.026]	0.637
**AX**	0.130	[-0.197, 0.457]	0.427
**X5**	-0.011	[-0.042, 0.020]	0.490
**fres**	0.108	[-0.116, 0.332]	0.338
**Predictor**	**Gender (Male)**		
**(Rrs) or (Xrs)**	**Beta Coefficient**	**95 % CI**	**P value**
**R5**	-0.644	NA	0.089
**R19**	-0.719	NA	0.004
**AX**	-1.558	NA	0.536
**X5**	0.316	NA	0.184
**fres**	-1.126	NA	0.516
**Predictor**	**Weight**		
**(Rrs) or (Xrs)**	**Beta Coefficient**	**95 % CI**	**P value**
**R5**	0.003	[-0.022, 0.027]	0.824
**R19**	-0.009	[-0.025, 0.007]	0.273
**AX**	0.098	[-0.061, 0.257]	0.220
**X5**	-0.006	[-0.021, 0.009]	0.433
**fres**	0.059	[-0.051, 0.169]	0.286
**Predictor**	**Height**		
**(Rrs) or (Xrs)**	**Beta Coefficient**	**95 % CI**	**P value**
**R5**	-4.645	[-8.036, -1.254]	0.008
**R19**	-4.043	[-6.211, -1.875]	<0.001
**AX**	-16.82	[-40.331, 6.684]	0.156
**X5**	2.102	[-0.101, 4.304]	0.060
**fres**	-12.838	[-28.942, 3.267]	0.115
**Predictor**	**BMI**		
**(Rrs) or (Xrs)**	**Beta Coefficient**	**95 % CI**	**P value**
**R5**	0.126	[0.032, 0.219]	0.009
**R19**	0.043	[-0.024, 0.110]	0.204
**AX**	0.963	[0.375, 1.550]	0.001
**X5**	-0.081	[-0.138, -0.023]	0.007
**fres**	0.649	[0.241, 1.056]	0.002

Table S4: *Multivariate linear regression for normative values.*

Predictors		Gender, Height, and BMI		
**(Rrs) or (Xrs)**	**Factor**	**Beta Coefficient**	**95 % CI**	**P value**
**R5**	**Gender**<br>**Height**<br>**BMI**	-0.135<br>-5.049<br>0.153	NA<br>[-9.800, -0.297]<br>[0.067, 0.238]	0.790<br>0.037<br><0.001
**R19**	**Gender**<br>**Height**<br>**BMI**	-0.339<br>-3.202<br>0.069	NA<br>[-6.485, 0.081]<br>[0.009, 0.128]	0.336<br>0.055<br>0.023
**AX**	**Gender**<br>**Height**<br>**BMI**	0.077<br>-23.384<br>1.064	NA<br>[-56.099, 9.332]<br>[0.473, 1.655]	0.982<br>0.156<br><0.001
**X5**	**Gender**<br>**Height**<br>**BMI**	0.196<br>1.951<br>-0.096	NA<br>[-1.174, 5.076]<br>[-0.153, -0.040]	0.559<br>0.214<br>0.001
**fres**	**Gender**<br>**Height**<br>**BMI**	0.470<br>-18.791<br>0.7163	NA<br>[-41.250, 3.667]<br>[0.310, 1.121]	0.844<br>0.098<br><0.001
**Predictors**		**Height, and BMI**		
**R5**	**Height**<br>**BMI**	-5.532<br>0.150	[-8.541, -2.523]<br>[0.067, 0.232]	<0.001<br><0.001
**R19**	**Height**<br>**BMI**	-4.414<br>0.063	[-6.516 -2.311]<br>[0.005, 0.120]	<0.001<br>0.033
**AX**	**Height**<br>**BMI**	-23.110<br>1.066	[-43.811, -2.408]<br>[0.497, 1.634]	0.029<br><0.001
**X5**	**Height**<br>**BMI**	2.649<br>-0.093	[0.663, 4.635]<br>[-0.147, -0.038]	0.010<br>0.001
**fres**	**Height**<br>**BMI**	-17.114<br>0.725	[-31.332, -2.896]<br>[0.334, 1.115]	0.019<br><0.001

Table S5: *Final Prediction equations for normal value FOT measures*

y	β0	B1	B2	RSE
**R5**	9.190	-5.532	0.150	1.012
**R19**	9.016	-4.413	0.063	0.706
**AX**	20.261	-23.110	1.066	6.959
**X5**	-3.573	2.649	-0.093	0.667
**fres**	26.196	-17.114	0.725	4.780

y= β0 + β1*Height + β2*BMI. Height: m; BMI: (Kg/m2) height range: 1.49–1.91 m; BMI range: 20.5–34.8 Kg/m2. The residual standard error (RSD) was reported from the regression model. Percentage predicted value (% pred) then estimated as the subject measured/ subject predicted value * 100 for the total population (310 healthy and disease groups).

***Table S6**: Correlation coefficients between FOT and symptoms, clinical parameters and blood biomarkers.*

	**Spo2**		**Respiratory Rate**		**MEWS**	
	coefficient	P value	coefficient	P value	coefficient	P value
**R5 ($kPa.s.L^{-1}$)**	-0.24	<0.001	0.16	0.006	0.04	0.489
**R19 ($kPa.s.L^{-1}$)**	-0.11	0.087	0.07	0.195	0.04	0.542
**R5-R19 ($kPa.s.L^{-1}$)**	-0.29	<0.001	0.2	<0.001	0.05	0.428
**X5**	0.29	<0.001	-0.26	<0.001	-0.04	0.493
**Ax (KPa/L)**	-0.32	<0.001	0.26	<0.001	0.07	0.250
**Fres (Hz)**	-0.35	<0.001	0.23	<0.001	0.11	0.081

	**Blood eosinophils count**		**CRP**		**BNP**	
	coefficient	P value	coefficient	P value	coefficient	P value
**R5 ($kPa.s.L^{-1}$)**	-0.12	0.038	0.18	0.001	0.18	0.062
**R19 ($kPa.s.L^{-1}$)**	-0.12	0.039	0.11	0.071	0.03	0.610
**R5-R19 ($kPa.s.L^{-1}$)**	-0.08	0.179	0.22	<0.001	0.16	0.009
**X5**	0.11	0.048	-0.21	<0.001	-0.18	0.003
**Ax (KPa/L)**	-0.08	0.153	0.23	<0.001	0.20	0.001
**Fres (Hz)**	0.04	0.489	0.26	<0.001	0.21	0.002

	**Breathlessness VAS score (mm)**		**Wheeze VAS score (mm)**	
	coefficient	P value	coefficient	P value
**R5 ($kPa.s.L^{-1}$)**	0.22	<0.001	0.21	<0.001
**R19 ($kPa.s.L^{-1}$)**	0.12	0.036	0.12	0.039
**R5-R19 ($kPa.s.L^{-1}$)**	0.26	<0.001	0.26	<0.001
**X5**	-0.28	<0.001	-0.25	<0.001
**Ax (KPa/L)**	0.33	<0.001	0.29	<0.001
**Fres (Hz)**	0.35	<0.001	0.31	<0.001

***Table S7**: Measures of FOT across symptoms, clinical parameters and blood biomarkers*

**Oxygen saturations (%)**						
**Total (n)**	R5 (kPa.s.L^{-1})	R19 (kPa.s.L^{-1})	R5-R19 (kPa.s.L^{-1})	X5	Ax (KPa/L)	Fres (Hz)
**Oxygen saturations (%)**						
**<92%** **n (12)**	6.125 4.405-8.652	3.775 3.015-5.350	2.020 1.170-3.695	-5.975 (-7.338)-(-2.828)	49.45 (29.19-73.31)	30.41 (25.23-32.77)
**92-96%** **n (126)**	5.430 4.105-7.415	3.710 2.905-4.902	1.675 1.062-2.445	-3.385 (-5.582)-(-2.312)	34.53 (17.70 -59.60)	26.50 (20.66-31.38)
**>96** **n (119)**	4.650 3.590-5.835	3.430 2.965-4.210	1.210 0.310-1.885	-2.270 (-3.895)-(-1.425)	20.00 (6.545-36.120)	22.48 (14.43-27.63)
**P value**	0.004	0.380	<0.001	<0.001	<0.001	<0.001
**Respiratory rate (breaths/min)**						
**<21** **n (217)**	5.000 (3.710 -6.910)	3.620 (2.900-4.710)	1.470 (0.610-2.220)	-2.900 (-5.090)-(-1.480)	26.600 (9.057-51.633)	24.20 (16.46-29.55
**21-29** **n (80)**	5.395 (4.285-7.095)	3.715 (2.978-4.730)	1.725 (1.073-2.550)	-3.595 (-6.130)-(-2.285)	40.02 19.68-70.33	27.64 (22.34-32.48
**30+** **n (6)**	3.945 (3.570-5.678)	3.585 (3.098-5.093)	0.4500 (0.150-0.8175)	-1.305 (-2.930)-(-1.165)	9.110 5.117-19.275	15.30 (12.51-22.37
**P value**	0.194	0.888	0.012	0.027	0.004	0.001
**Modified early warning score**						
**0-2** **n (182)**	5.060 (3.710-6.765)	3.580 (2.905-4.665)	1.5350 (0.5675-2.2125)	-2.840 (-4.957)-(-1.528)	26.58 (10.26 -50.50)	24.12 (17.10-29.36)
**3** **n (28)**	4.770 (3.395-5.822)	3.120 (2.572-4.030)	1.440 (0.665-2.180)	-3.190 (-3.825)-(-1.675)	29.36 (9.73-44.07)	27.35 (17.34-32.11)
**>3** **n (55)**	5.390 (4.130-7.505)	3.650 (3.035-5.310)	1.400 (0.855-2.520)	-3.430 (-5.695) -(-1.900)	31.96 (16.30-66.64)	25.02 (19.97-32.59)
**P value**	0.130	0.034	0.731	0.494	0.350	0.105
**Eosinophil count, x10^9/L**						
**<0.30 × 109/L** **N (245)**	5.130 (3.920-7.340)	3.740 (2.950-5.000)	1.520 (0.700-2.480)	-3.000 (-6.060) -(-1.620)	30.19 (11.72-65.79)	24.71 (18.14-30.62)
**≥0.30 × 109/L** **n (56)**	5.005 (3.748-6.037)	3.430 (2.915-4.185)	1.345 (0.825-1.883)	-3.095 (-3.975)-(-1.502)	27.79 (13.13-43.65)	24.23 (17.57-29.32)
**P value**	0.149	0.151	0.246	0.258	0.305	0.953
**Brain natriuretic peptide (ng/l)**						
**<400 (ng/L)** **n (221)**	5.090 (3.870-6.980)	3.590 (2.940-4.680)	1.480 (0.660-2.330)	-2.990 (-5.530)-(-1.560)	27.61 (11.70-58.78)	24.36 (17.12-30.41)
**≥400 (ng/L)**	5.690	4.065	1.6250	-4.045	36.50	26.56

**n (34)**	(4.853-6.825)	(3.265-5.447)	(0.9475-2.1800)	(-7.537)-(-2.400)	(23.01-73.62)	(23.30-32.59)
**P value**	0.210	0.233	0.604	0.019	0.076	0.026
**C-reactive protein (mg/L)**						
**<40 (mg/L) n (217)**	5.090 (3.710-6.840)	3.510 (2.940-4.870)	1.470 (0.640-2.300)	-3.020 (-5.600)-(-1.510)	29.34 (10.06-58.04)	24.03 (17.16-30.01)
**≥40 (mg/L) n (77)**	5.14 (4.07-7.35)	3.810 (2.970-4.650)	1.640 (0.830-2.770)	-3.28 (-5.90)-(-1.84)	32.52 (13.86-59.08)	25.77 (18.58-30.63)
**P value**	0.440	0.715	0.254	0.334	0.329	0.319
**Breathlessness VAS score (mm)**						
**0-25**	4.020 (3.365 - 5.275)	3.280 (2.775 - 3.935)	0.7400 (0.240 - 1.500)	-1.580 (-2.515) – (-1.135)	8.79 (3.99-20.10)	17.83 (12.84-23.16)
**26-50**	5.350 (3.650 - 7.140)	3.530 (2.970 - 4.870)	1.470 (0.820-2.220)	-3.630 (-5.020)-(-1.840)	33.06 (15.58-66.82)	23.84 (22.43-31.29)
**51-75**	5.600 (4.525 - 7.308)	3.845 (3.112 - 5.025)	1.770 (0.975-2.607)	-3.385 (-5.582)-(-2.030)	33.02 (17.74-55.14)	26.51 (19.50-31.05)
**76-100**	5.290 (4.110 - 7.395)	3.810 (2.870 - 4.930)	1.660 (1.015-2.490)	-3.670 (-6.505)-(-2.340)	40.74 (19.68-70.11)	26.83 (22.00-31.99)
**P value**	<0.001	0.010	<0.001	<0.001	<0.001	<0.001
**Wheeze VAS score (mm)**						
**0-25**	4.830 (3.610 - 6.090)	3.560 (2.940 - 4.250)	1.210 (0.420 - 1.840)	-2.370 (-3.850)-(-1.300)	20.0 (6.57)-(38.41)	22.53 (13.95-26.91)
**26-50**	5.040 (4.160 - 7.060)	3.530 (2.895 - 4.830)	1.470 (0.935 - 2.245)	-2.610 (-5.975)-(-2.025)	31.39 (14.93)-(58.62)	25.91 (18.69-30.28)
**51-75**	5.140 (4.120 - 7.555)	3.430 (3.050 - 4.895)	1.700 (1.130 - 2.585)	-3.580 (-6.635)-(-2.085)	34.53 (19.32)-(62.70)	26.37 (20.07-31.56)
**76-100**	5.555 (4.460 - 7.440)	3.935 (3.010 - 4.995)	1.775 (0.980 - 2.635)	-3.720 (-5.860)-(-2.062)	41.03 (18.88)-(73.52)	27.01 (22.18-32.00)
**P value**	0.016	0.354	<0.001	<0.001	<0.001	<0.001

Figure S1: *Differences in R5-19 and Ax in blood biomarkers*

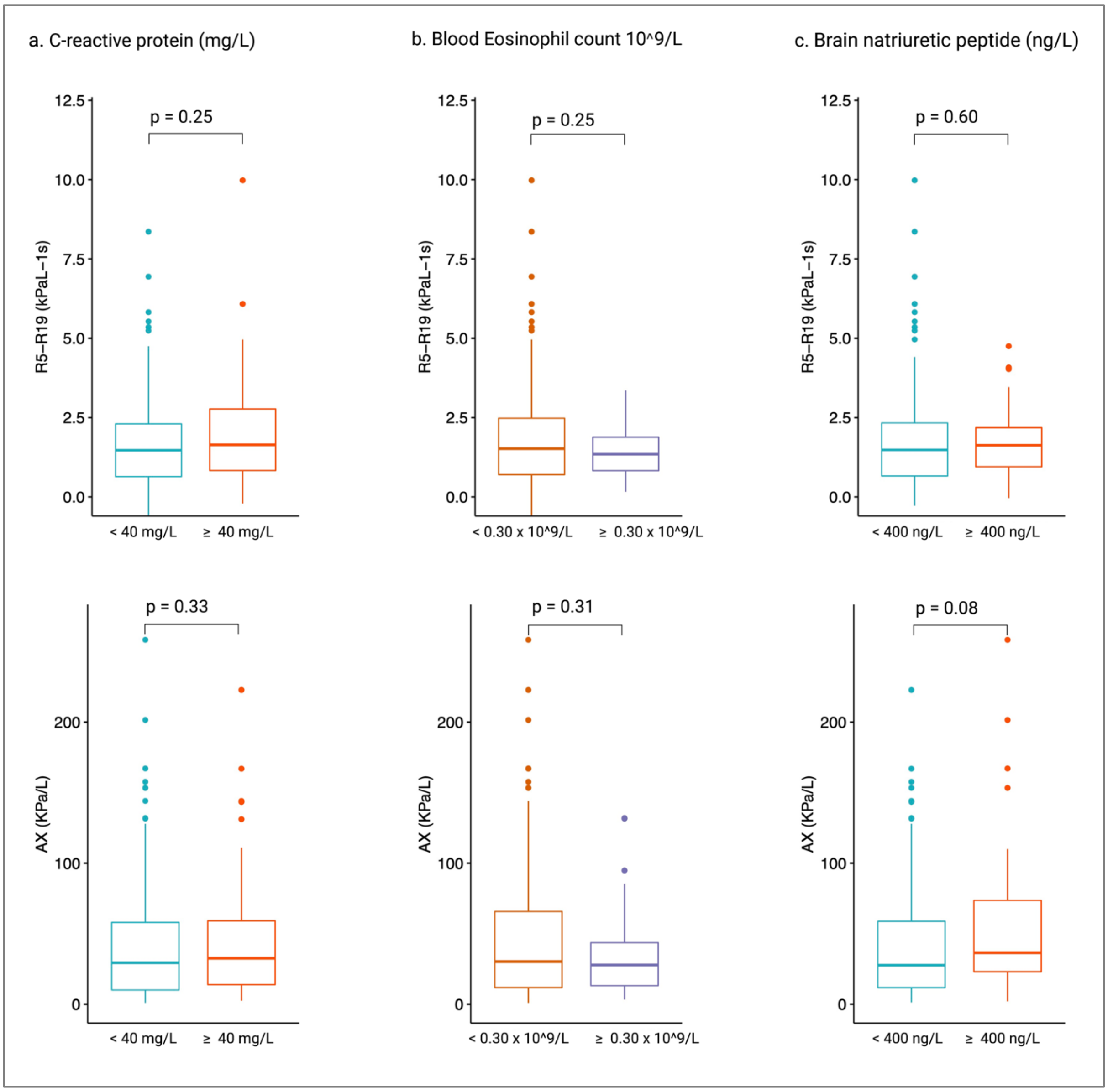

Table S8: Demographic description of acute patients who did and did not attend follow up recovery visit.

	Attended Recovery Visits	Did not attend recovery visit
**Age, years**	62.4 ±17.7	57.8 ±18.8
**Male Sex, n (%)**	41 (65.1)	100 (49.5%)
**Height, m**	1.70 ±0.09	1.68 ±0.10
**Weight, Kg**	83.5 ±19.0	82.6 ±25.9
**BMI, Kg/m^2**	29.0 ±6.1	29.3 ±8.7
**Diagnosis**		
**Asthma**	19 (30%)	61 (31%)
**COPD**	20 (32%)	55 (28%)
**Pneumonia**	13 (21%)	49 (25%)
**Heart Failure**	11 (17%)	35 (18%)

Figure S2: Paired changes in R5-R19 and Ax across different diseases

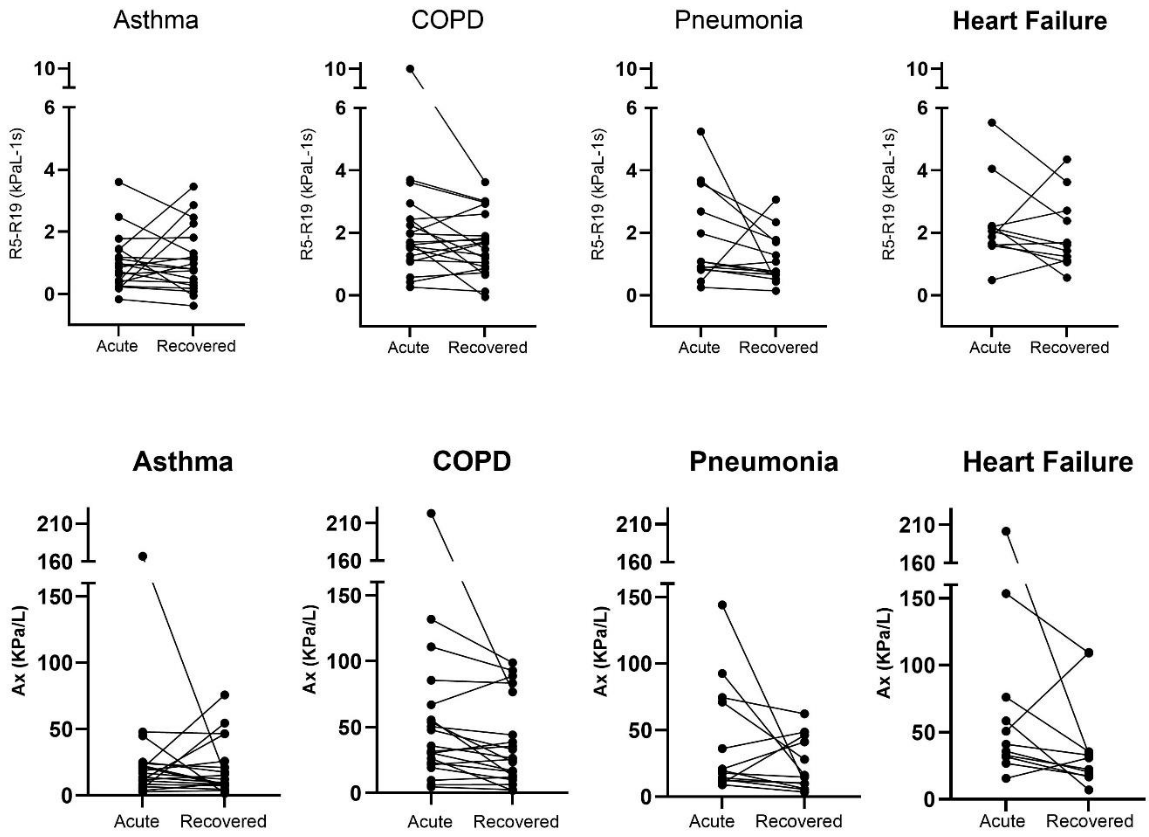

1. Oostveen E. Respiratory impedance in healthy subjects: baseline values and bronchodilator response. *Eur Respir J* 2013; **42**.
